# Practices and Perceptions of Moroccan Dentists Regarding Flexible Removable Dentures: A Cross-Sectional Study in the Rabat-Salé-Kénitra Region

**DOI:** 10.7759/cureus.110626

**Published:** 2026-06-10

**Authors:** Ayah Krichal, Ismail Akhatou, Nadia Merzouk, Hassnae Benyahia, Leila Fajri

**Affiliations:** 1 Department of Removable Prosthodontics, Faculty of Dental Medicine, Mohammed V University, Rabat, MAR

**Keywords:** aesthetics, edentulism, flexible resin, questionnaire, removable flexible denture

## Abstract

Background and aim

Flexible removable dentures are increasingly used in partial prosthetic rehabilitation because of their aesthetic appeal and mechanical flexibility. However, their limitations should be considered carefully, and their use should be restricted to selected clinical situations. This study aimed to assess the clinical practices and perceptions of Moroccan private-sector dentists in the Rabat-Salé-Kénitra region regarding the indications, advantages, disadvantages, and clinical use of flexible removable dentures, as well as the association between their use and participants’ demographic and professional characteristics.

Materials and methods

This cross-sectional study used an anonymous online questionnaire developed in Google Forms (Google LLC, Mountain View, CA, USA) and administered between November 2023 and February 2024 to licensed private-sector dentists practicing in the Rabat-Salé-Kénitra region. Qualitative variables were expressed as counts and percentages (n (%)). Associations between the use of flexible removable dentures and participants’ demographic and professional characteristics were analyzed using the chi-square test or Fisher’s exact test. A p-value < 0.05 was considered statistically significant.

Results

A total of 101 private-sector dentists participated in the study. Flexible removable dentures were used in clinical practice by 86 (85.1%) participants, and 82 (98.8%) clinicians used them mainly for partial edentulism. 48 (55.2%) practitioners considered flexible dentures suitable as definitive prostheses. In cases of extensive partial edentulism, 61 (62.2%) participants preferred metal-based removable partial dentures combined with acrylic resin. Valplast (Valplast International Corp., Westbury, NY, USA) was the most commonly used material by 56 (67.5%) participants, and 68 (71.6%) practitioners reported favorable satisfaction with flexible dentures.

Conclusions

Flexible removable dentures appear to be widely used among Moroccan private-sector dentists, particularly for the management of partial edentulism and aesthetically demanding situations. However, their use remains less favored in extensive edentulous cases. These findings highlight the need for greater emphasis in undergraduate and continuing dental education regarding the indications, limitations, and appropriate clinical application of flexible removable dentures.

## Introduction

Patients once had relatively modest expectations regarding the functional and aesthetic outcomes of conventional removable prostheses. However, with increased public awareness, these expectations have risen significantly, particularly concerning aesthetics. As a result, many patients now tend to avoid removable partial dentures (RPDs), often associating them with aging [1], especially given that their management as definitive prostheses is complex and may require pre-prosthetic surgical interventions, careful planning of the path of insertion and removal, the use of resilient lining materials to improve adaptation, and may still compromise aesthetics due to visible clasps [2].

Polymethyl methacrylate (PMMA) resin, the material of choice for denture bases, presents several advantages, including ease of manipulation and repair, low cost, good patient acceptance, stability in the oral environment, and satisfactory aesthetic properties. However, its mechanical performance remains suboptimal, with low flexural and impact strength and limited fatigue resistance, which may lead to fractures during use or when accidentally dropped [3]. In addition, the presence of metal clasps in conventional prostheses may compromise aesthetics, particularly in visible areas. Furthermore, some patients may present hypersensitivity or allergic reactions to metallic components, which can limit their use [4,5].

Flexible dentures represent an alternative prosthetic design that allows for optimal flange height and thickness. They are also commonly known as “metal-free” or “clasp-free” dentures. These prostheses are made from nylon-based thermoplastic materials, which preserve function while offering improved aesthetics. A variety of commercial products are available, including Valplast (Valplast International Corp., Westbury, NY, USA), Flexite (Flexite Company, Inc., Islandia, NY, USA), and Lucitone (Dentsply Sirona, York, PA, USA); among these, Valplast and Lucitone are monomer-free, which makes them suitable for patients with acrylic allergies [6].

Flexible denture materials offer several advantages that contribute to their growing use. Their translucency allows them to blend with surrounding tissues, making them highly aesthetic, especially given their absence of visible clasps. They are lightweight, flexible, and comfortable, with good biocompatibility because they are free of monomers and metals, reducing the risk of allergic reactions. Their elasticity provides a stress-breaking effect, improving load distribution and preserving supporting tissues while minimizing sore spots commonly associated with rigid acrylic bases [7,8].

However, because of their technique-sensitive processing and material-related limitations, flexible dentures require careful case selection and strict adherence to fabrication protocols. Although they offer favorable aesthetics and flexibility, concerns have been reported regarding long-term color stability, maintenance, and biomechanical performance. Their lower rigidity compared with that of PMMA may compromise support in extensive edentulous situations, while surface wear and tooth debonding may occur over time. In addition, certain clinical situations, such as deep overbite, distal extension cases, or flabby tissues, may contraindicate their use [6,9].

Despite its growing popularity, this prosthesis has not been adequately addressed in prosthodontics teaching programs at Moroccan dental faculties, and its use, particularly in the management of specific cases of partial edentulism, is variably adopted in private-sector clinical practice. Accordingly, this study aimed to evaluate the use, clinical practices, and perceptions of Moroccan private-sector dentists in the Rabat-Salé-Kénitra region regarding the advantages, limitations, indications, frequency of use, and clinical implementation of flexible removable dentures in routine dental practice, as well as the association between their use and participants’ demographic and professional characteristics.

## Materials and methods

Study design and setting

This cross-sectional study was conducted from November 28, 2023, to February 17, 2024. The study targeted private practitioners and clinicians in the Rabat-Salé-Kénitra region.

Sample size

The sample size was calculated using an online sample size calculator for cross-sectional studies (Cleveland Clinic) to estimate a population proportion with a specified level of precision. Assuming a 95% confidence level (α = 0.05), an expected proportion of 0.349 (34.9%), representing the estimated proportion of clinicians using flexible prostheses based on previous findings by Elfaidy et al. [10] in their survey of Libyan dental clinicians’ attitudes toward flexible dentures in 2022, and a margin of error of 10%, the minimum required sample size was estimated at 88 participants. A total of 101 dentists were included in the study. The chosen margin of error was considered acceptable given the study’s descriptive nature and the practical constraints of participant recruitment. The final sample size exceeded the minimum requirement, thereby enhancing the precision of the estimates.

Inclusion and exclusion criteria

Dentists were considered eligible if they were actively practicing in the Rabat-Salé-Kénitra region at the time of the study and had completed academic or private training, either in Morocco or abroad, allowing them to deliver comprehensive dental care. Participation was voluntary and based on informed consent, as evidenced by completion of the questionnaire. Dentists who were not practicing in the specified region or who failed to complete the demographic section of the questionnaire were excluded from the study.

Ethical considerations

This study was conducted in accordance with the principles of the Declaration of Helsinki and local recommendations. Informed consent was implied through voluntary completion of the questionnaire after participants had been informed about the study’s purpose and procedures. Participation was voluntary, and participants’ confidentiality and anonymity were maintained throughout the research process. No sensitive personal data, biological samples, or invasive procedures were involved.

Questionnaire

The authors developed an original questionnaire in French, the primary language of dental education in Morocco, based on a review of the relevant literature and current dental practices (Appendix A) [2,4,8,10]. Before distribution, the instrument was reviewed by prosthodontic specialists to assess the clarity, relevance, and comprehensibility of its items. The questionnaire addressed the use of flexible dentures, including their advantages, indications, and the materials employed. It consisted of 23 items and was organized into two sections. The first section collected demographic data, such as age, gender, city of practice, years of experience, university of graduation, and participation in continuing education. The second section explored Moroccan dentists’ attitudes toward flexible dentures, focusing on their use, indications, advantages, and the available materials. The questionnaire was self-administered online via Google Forms (Google LLC, Mountain View, CA, USA) and distributed using a convenience and snowball sampling method through professional dental social media groups and direct contact. The survey took approximately three to five minutes to complete, and no personally identifiable data was collected. Only the questionnaire items relevant to the objectives of the present study were included in the final statistical analysis and presented in the tables.

Statistical analysis

The statistical analysis was performed using jamovi (version 2.7.8; The jamovi Project, Sydney, Australia). All qualitative variables (age, sex, city of practice, country of graduation, years of clinical practice, professional status, city of practice, postgraduate continuing education in prosthodontics, the use of flexible removable dentures in the practice, frequency of use of flexible removable dentures, type of flexible removable dentures used, purpose of flexible denture use, etc.) were expressed as n (frequency (%)). Response rates varied across the second section of the questionnaire items because some participants did not answer all the questions. Missing responses were excluded from the analysis on an item-by-item basis, and percentages were calculated based on the number of valid responses for each questionnaire item. Some participants who did not currently use flexible removable dentures may nevertheless have answered selected questions based on previous clinical experience with these prostheses. Associations between the use of flexible removable dentures and participants’ demographic and professional characteristics were analyzed using the chi-square test or Fisher’s exact test when one or more expected cell counts were less than 5. A p-value < 0.05 was considered statistically significant. Where appropriate, response categories were grouped to facilitate analysis and interpretation.

## Results

The study included 101 private-sector dentists practicing in the Rabat-Salé-Kénitra region. Among the participants, 56 (55.4%) were male, 72 (71.3%) were aged 20-30 years, and 80 (79.2%) had 1-10 years of clinical experience. A total of 81 (80.2%) participants were general dental practitioners, and 42 (41.6%) had received continuing education in prosthodontics (Table 1).

**Table 1:** Demographic and professional characteristics of the participants Data are presented as n (%).

Variables	N = 101
Age categories (years)	
20-30	72 (71.3)
31-40	24 (23.7)
41-50	3 (3.0)
Above 50	2 (2.0)
Sex	
Female	45 (44.6)
Male	56 (55.4)
Country of graduation	
Moroccan degree (public or private faculty)	85 (84.2)
Foreign degree	16 (15.8)
Years of clinical practice (years)	
1-10	80 (79.2)
10-20	19 (18.8)
20-30	2 (2.0)
Professional status	
General dentist	81 (80.2)
Specialist (non-prosthodontist)	19 (18.8)
Prosthodontist	1(1.0)
City of practice	
Kénitra	20 (19.8)
Rabat	38 (37.6)
Salé	35 (34.7)
Témara	8 (7.9)
Postgraduate continuing education in prosthodontics	
Yes	42 (41.6)
No	59 (58.4)

A total of 86 (85.1%) participants reported using flexible removable dentures in their clinical practice, although the frequency of use was mainly occasional (44 (51.8%)). Flexible dentures were predominantly used for partial edentulism (82 (98.8%)), while their use in complete edentulism remained very limited. A total of 48 (55.2%) practitioners considered flexible dentures suitable as definitive prostheses (Table 2).

**Table 2:** Use, indications, and perceptions of flexible removable dentures among participantsong participants Data are presented as n (%). Percentages were calculated based on valid responses for each item; missing responses were excluded from the analysis. Some participants who did not currently use flexible removable dentures may have answered selected questions based on previous clinical experience with these prostheses. RPD, removable partial denture

Variables	n (%)	95% CI
Use of flexible removable dentures in clinical practice (N = 101)		
Yes	86 (85.1)	76.69-91.44
No	15 (14.9)	8.55-23.31
If yes, frequency of use of flexible removable dentures (N = 85)		
Sometimes	44 (51.8)	40.66-62.74
Rarely	26 (30.6)	21.04-41.53
Often	15 (17.6)	10.22-27.43
Type of flexible removable dentures used (N = 83)		
Partial dentures	82 (98.8)	93.46-99.97
Partial and complete dentures	1 (1.2)	0.03-6.53
Clinical purpose of flexible denture use (N = 87)		
Temporary dentures	31 (35.6)	25.64-46.62
Definitive dentures	48 (55.2)	44.12-65.85
Both temporary and definitive dentures	8 (9.2)	4.05-17.32
Dental arch of flexible denture use (N = 87)		
Maxillary	37 (42.5)	31.98-53.59
Mandibular	9 (10.4)	4.84-18.73
Both arches	41 (47.1)	36.32-58.13
Preferred prosthetic rehabilitation in cases of extensive partial edentulism (N = 98)		
Flexible RPD	3 (3.1)	0.63-8.69
Conventional acrylic RPD	25 (25.5)	17.23-35.31
A combination of metal framework and flexible resin	9 (9.2)	4.28-16.72
A combination of metal framework and acrylic resin	61 (62.2)	51.88-71.84
In the presence of bilateral undercuts (N = 98)		
Pre-prosthetic surgery is essential, regardless of the type of resin used	55 (56.1)	45.73-66.13
Flexible resin may be preferred over acrylic resin	43 (43.9)	33.86-54.27
Use of flexible dentures in patients with bruxism (N = 98)		
Yes	55 (56.1)	45.73-66.13
No	43 (43.9)	33.86-54.27
Type of flexible denture base material used (N = 83)		
Valplast	56 (67.5)	56.30-77.35
Flexite	14 (16.9)	9.54-26.68
Valplast and Flexite	2 (2.4)	0.29-8.43
Do not know	11 (13.2)	6.80-22.48
Patient complaints related to flexible dentures (N = 92)		
Mild	22 (23.9)	15.63-33.94
Moderate	61 (66.3)	55.69-75.83
Severe	9 (9.8)	4.57-17.76
Satisfaction level toward flexible dentures (N = 95)		
Favorable	68 (71.6)	61.40-80.36
Unfavorable	27 (28.4)	19.63-38.60

Regarding preferred prosthetic rehabilitation for extensive partial edentulism, 61 (62.2%) participants favored metal-based RPDs combined with acrylic resin, while only 3 (3.1%) preferred fully flexible RPDs. In the presence of bilateral undercuts, 55 (56.1%) participants considered pre-prosthetic surgery necessary regardless of the material used (Table 2).

A total of 55 (56.1%) participants reported using flexible dentures in patients with bruxism. Valplast was the most commonly used flexible denture material (56 (67.5%)), followed by Flexite (14 (16.9%)). Favorable satisfaction toward flexible dentures was reported by 68 (71.6%) practitioners, while moderate patient complaints were the most frequently reported (61 (66.3%)) (Table 2).

No statistically significant associations were found between the use of flexible removable dentures and participants’ demographic or professional characteristics, including age category, sex, university of graduation, years of professional experience, professional status, city of practice, and continuing education in prosthodontics (p > 0.05) (Table 3).

**Table 3:** Association between participants’ demographic and professional characteristics and the use of flexible removable dentures * Chi-square test was used for comparisons. ** Fisher’s exact test was used because one or more expected cell counts were less than 5.

Variables	No, n (%)	Yes, n (%)	χ²	p-value
Age categories^**^			-	1.000
20-30 years	11 (15.3)	61 (84.7)		
31-40 years	4 (16.7)	20 (83.3)		
41-50 years	0 (0.0)	3 (100.0)		
Above 50 years	0 (0.0)	2 (100.0)		
Sex^*^			0.0318	0.858
Female	7 (15.6)	38 (84.4)		
Male	8 (14.3)	48 (85.7)		
Country of graduation^**^			-	1.000
Moroccan university	13 (15.3)	72 (84.7)		
Foreign university	2 (12.5)	14 (87.5)		
Years of clinical practice^**^			-	0.804
1-10	13 (16.3)	67 (83.8)		
10-20	2 (10.5)	17 (89.5)		
20-30	0 (0.0)	2 (100.0)		
Professional status^**^			-	0.206
General dentist	12 (14.8)	69 (85.2)		
Specialist (non-prosthodontist)	2 (10.5)	17 (89.5)		
Prosthodontist	1 (100.0)	0 (0.0)		
City of practice^**^			-	0.295
Kénitra	2 (10.0)	18 (90.0)		
Rabat	9 (23.7)	29 (76.3)		
Salé	3 (8.6)	32 (91.4)		
Témara	1 (12.5)	7 (87.5)		
Postgraduate continuing education in prosthodontics^*^			0.0182	0.893
No	9 (15.3)	50 (84.7)		
Yes	6 (14.3)	36 (85.7)		

## Discussion

This survey aimed to evaluate the use, clinical practices, and perceptions of private-sector dentists in the Rabat-Salé-Kénitra region regarding flexible removable dentures, including their indications, frequency of use, application in patients with bruxism, and management of bilateral undercuts, as well as the association between their use and participants’ demographic and professional characteristics. The findings revealed a high prevalence of flexible denture use among Moroccan practitioners, particularly in the management of partial edentulism and aesthetically demanding situations. However, practitioners remained cautious regarding their use in extensive edentulous cases, where metal-based removable prostheses were preferred, and opinions were divided regarding their use in patients with bruxism.

Most participants were general dental practitioners (80.2%) with one to 10 years of professional experience (79.2%), and the majority reported using flexible removable dentures in their clinical practice. These findings suggest that flexible dentures are increasingly incorporated into daily prosthodontic practice despite limited formal training in this field. Similar findings were reported by Polyzois et al. [2], who found that general practitioners prescribed flexible dentures more frequently than specialists. Likewise, Elfaidy et al. [10] reported favorable perceptions of flexible dentures among Libyan dentists despite the absence of dedicated university teaching concerning flexible prostheses. Comparable observations were reported by Rozano et al. [4] in Malaysia, where practitioners demonstrated increasing awareness and clinical use of thermoplastic removable prostheses. These findings may reflect growing international interest in flexible prosthetic solutions due to their aesthetic advantages. The predominance of younger practitioners among users may also reflect greater exposure to newer prosthodontic materials through professional networking platforms and manufacturer-driven educational resources.

Our findings demonstrated that flexible dentures were primarily indicated for partial edentulism, with 98.8% of participants reporting their use in such patients. In contrast, their use in complete edentulism remained very limited. These results are consistent with those reported by Elfaidy et al. [10], who found that flexible dentures were mainly prescribed for partially edentulous patients and for patients presenting repeated fractures of conventional acrylic dentures. This preference is likely related to the superior aesthetics of flexible dentures, particularly the absence of visible metal clasps, as well as their ability to engage soft-tissue undercuts with minimal tooth preparation. Conversely, their limited rigidity may reduce their suitability for complete edentulism, where optimal support, stability, and load distribution are essential [6,7].

Regarding the clinical use of flexible dentures, 55.2% of practitioners considered them suitable as definitive prostheses. Similar variations were described by Polyzois et al. [2], who reported that flexible dentures were mainly used as provisional restorations in Greece, whereas Croatian practitioners more frequently considered them definitive prostheses. Elfaidy et al. [10] also reported nearly equivalent use of flexible dentures for both temporary and permanent rehabilitation. These differences between practitioners and countries may be related to variations in clinical experience, prosthodontic philosophy, continuing education, and patient expectations. They may also reflect the absence of universally accepted clinical guidelines regarding the long-term use of flexible dentures. Consequently, clinicians often rely on personal experience and local practice patterns when determining whether these prostheses should serve as temporary or definitive treatment options.

In cases of extensive partial edentulism, most participants in our survey preferred metal-based RPDs combined with acrylic resin. These findings suggest that Moroccan dentists remain cautious regarding the use of fully flexible dentures in extensive edentulous situations. Fueki et al. [1] emphasized that flexible dentures should be carefully indicated for extensive edentulous spans due to their biomechanical limitations. Consequently, reinforcement with metallic frameworks may be necessary to improve stress distribution and prosthesis stability in large-span rehabilitations. These findings indicate that clinicians continue to prioritize biomechanical stability over aesthetic considerations when rehabilitating extensive edentulous spaces.

Valplast was the most frequently used thermoplastic material in the present study, followed by Flexite. However, 13.2% of participants were unable to identify the commercial name of the material used. Similar findings were reported by Elfaidy et al. [10], who observed limited knowledge among practitioners regarding the commercial materials used for flexible dentures. This observation may reflect insufficient continuing education concerning flexible denture materials and their mechanical properties. The inability of some participants to identify the commercial material used may be clinically relevant, as different thermoplastic systems can exhibit distinct mechanical properties, processing requirements, and repair characteristics that may influence treatment planning and long-term clinical performance.

In the presence of bilateral undercuts, 56.1% of participants considered pre-prosthetic surgery essential regardless of the material used. These results indicate that Moroccan dentists remain divided regarding the management of bilateral undercuts using flexible dentures. Saketharaman et al. [8] similarly reported that flexible dentures were frequently indicated for patients with undercuts and for those allergic to acrylic resin. The flexibility and adaptability of thermoplastic resins may therefore provide a conservative alternative in selected clinical situations where extensive surgical correction may otherwise be required. Nevertheless, the divided responses observed in the present survey suggest a lack of consensus regarding the extent to which flexible materials can compensate for anatomical limitations without compromising prosthesis retention and stability.

A total of 56.1% of participants reported using flexible dentures in patients with bruxism. However, the literature remains controversial regarding this indication. Although flexible dentures exhibit greater flexibility than conventional acrylic prostheses [6], excessive occlusal loading associated with bruxism may compromise their long-term stability due to deformation and material fatigue. The relatively high proportion of practitioners reporting their use in patients with bruxism may reflect confidence in the material’s flexibility and fracture resistance. Nevertheless, flexibility alone does not necessarily guarantee favorable biomechanical performance under excessive occlusal loading, which may explain the absence of consensus among practitioners. Therefore, careful case selection remains essential when considering flexible dentures [9].

The long-term clinical performance of flexible removable dentures remains a subject of debate. Although these prostheses offer aesthetic and comfort-related advantages, several authors have highlighted challenges related to maintenance, repair, relining, and adjustment procedures [6,7,9]. Unlike conventional acrylic dentures, thermoplastic materials may be more difficult to modify after fabrication, complicating long-term follow-up and management. In addition, concerns have been raised regarding their long-term biomechanical performance and the limited evidence supporting their use in certain clinical situations [6,9]. These limitations should be carefully considered when selecting flexible dentures as definitive treatment options and reinforce the importance of evidence-based case selection.

Overall, 71.6% of practitioners expressed favorable satisfaction toward flexible dentures. Regarding patient complaints, moderate complaints were the most frequently reported. The favorable perception observed in this survey may be explained by the superior aesthetics, flexibility, lightweight structure, and absence of visible metallic clasps associated with thermoplastic dentures. Despite this favorable perception, the predominance of moderate patient complaints suggests that aesthetic benefits do not completely eliminate functional and maintenance-related concerns. This finding highlights the importance of balancing patient expectations with the biomechanical limitations of flexible prosthetic materials.

Limitations of the present study include a relatively small sample size, restriction to private-sector dentists practicing in the Rabat-Salé-Kénitra region, and the use of a self-administered online questionnaire, which may have introduced response bias. In addition, the predominance of younger practitioners and dentists with fewer years of clinical experience may limit the generalizability of the findings, as these groups may have been more likely to participate in an online survey. Furthermore, although the questionnaire was developed from the literature and reviewed by prosthodontic specialists, formal psychometric validation and reliability testing were not performed, which may affect the robustness of the survey instrument. Despite these limitations, the study provides valuable insights into the current use, indications, and perceptions of flexible removable dentures among Moroccan dentists, highlighting the growing interest in thermoplastic prosthetic materials and the need for enhanced continuing education and clinical guidelines regarding their appropriate use.

## Conclusions

Within the limitations of the present study, flexible removable dentures appear to be widely used among Moroccan private-sector dentists in the Rabat-Salé-Kénitra region, particularly for managing partial edentulism and aesthetically demanding cases. However, their use remains less favored in extensive edentulous cases, consistent with concerns about the biomechanical limitations of flexible prosthetic materials.

These findings highlight the need for greater emphasis in undergraduate dental curricula and continuing professional education on the indications, limitations, maintenance requirements, and long-term clinical performance of flexible removable dentures. Further multicenter studies with larger, more representative samples are recommended to better evaluate their clinical use, long-term outcomes, and perceptions among Moroccan dentists and to support the development of evidence-based clinical guidelines for their appropriate application.

## Appendices

Appendix A: Study questionnaire 

**Table 4:** French and English versions of the questionnaire used in the study

Original French version of the questionnaire	Translated English version of the questionnaire
1. Tranches d’âge	1. Age categories
Une seule réponse possible	Single choice
20-30 ans	20-30 years
31-40 ans	31-40 years
41-50 ans	41-50 years
Plus de 50 ans	Above 50 years
2. Genre	2. Sex
Une seule réponse possible	Single choice
Femme	Female
Homme	Male
3. Université Diplômante	3. University of graduation
Une seule réponse possible	Single choice
Faculté de médecine dentaire de Rabat	Faculty of Dental Medicine of Rabat
Faculté de médecine dentaire de Casablanca	Faculty of Dental Medicine of Casablanca
Université privée marocaine	Moroccan private university
Université étrangère	Foreign university
4. Expérience professionnelle	4. Professional experience
Une seule réponse possible	Single choice
1-10 ans	1-10 years
10-20 ans	10-20 years
20-30 ans	20-30 years
Plus de 30 ans	More than 30 years
5. Domaine d’exercice	5. Professional status
Plusieurs réponses possibles	Multiple choices
Chirurgien-dentiste (omnipraticien)	General dentist
Spécialiste en chirurgie maxillo-faciale	Specialist in maxillofacial surgery
Spécialiste en prosthodontie	Prosthodontist
Spécialiste en implantologie	Implantologist
Spécialiste en orthopédie dento-faciale	Orthodontist
Spécialiste en odontologie chirurgicale	Specialist in oral surgery
Autre	Other
6. Ville d’exercice	6. City of practice
Une seule réponse possible	Single choice
Rabat	Rabat
Salé	Salé
Kénitra	Kénitra
Autre	Other
7. Avez-vous suivi une formation continue en prothèse au cours de votre carrière ?	7. Have you attended any post-graduate continuing education courses in prosthodontics during your career?
Une seule réponse possible	Single choice
Oui	Yes
Non	No
8. Utilisez-vous la prothèse adjointe flexible dans votre cabinet ?	8. Do you use flexible removable dentures in your practice?
Une seule réponse possible	Single choice
Oui	Yes
Non	No
9. Si oui, à quelle fréquence par rapport à la résine acrylique ?	9. If yes, how frequently do you use flexible dentures compared with acrylic resin dentures?
Une seule réponse possible	Single choice
Souvent	Often
Parfois	Sometimes
Rarement	Rarely
10. Si oui, dans quel cas indiquez-vous la résine flexible ?	10. If yes, in which situations do you indicate flexible resin dentures?
Plusieurs réponses possibles	Multiple choices
Tous les édentements partiels	All partial edentulism cases
Les édentements partiels de petite étendue	Small partial edentulism
Les édentements partiels de moyenne étendue	Moderate partial edentulism
Les édentements partiels de grande étendue	Extensive partial edentulism
Les édentements complets	Complete edentulism
11. Vous optez pour la résine flexible dans le cadre de :	11. Flexible resin dentures are mainly used as:
Plusieurs réponses possibles	Multiple choices
Prothèse provisoire	Temporary prosthesis
Prothèse transitoire	Transitional prosthesis
Prothèse d’usage	Definitive prosthesis
12. Quelles sont vos raisons d’utiliser la prothèse amovible flexible ?	12. What are your reasons for using flexible removable dentures?
Plusieurs réponses possibles	Multiple choices
Choix propre du patient	Patient’s own choice
Satisfaction des patients ayant bénéficié de ce type de prothèse	Patient satisfaction
Les patients âgés (utilisation simple)	Ease of use in elderly patients
Visibilité de crochets sur les canines maxillaires	Visibility of metal clasps on maxillary canines
Autre	Other
13. D’après votre expérience, quels sont les principaux avantages de la résine flexible ?	13. According to your experience, what are the main advantages of flexible resin dentures?
Plusieurs réponses possibles	Multiple choices
S’affranchir de crochets métalliques	Absence of metal clasps
Longévité de la prothèse	Prosthesis longevity
Absence de doléances	Reduced patient complaints
Intégration fonctionnelle aisée	Easier functional integration
Meilleure biocompatibilité	Better biocompatibility
Résistance aux fractures	Fracture resistance
14. Dans le cas où vous n’utilisez pas la prothèse adjointe flexible, quelles en sont les raisons ?	14. If you do not use flexible removable dentures, what are the reasons?
Plusieurs réponses possibles	Multiple choices
Coût	Cost
Instabilité de l’occlusion	Occlusal instability
Réparation difficile	Difficult of repair
Autre	Other
15. Au laboratoire, quels sont les matériaux que vous utilisez pour l’élaboration de la prothèse adjointe flexible ?	15. Which materials are used in your laboratory for the fabrication of flexible removable dentures?
Plusieurs réponses possibles	Multiple choices
La résine Valplast (Unival)	Valplast® resin (Unival)
La résine Lucitone FRS (Dentsply International)	Lucitone FRS® resin (Dentsply International)
La résine Flexite (Flexite Company)	Flexite® resin (Flexite Company)
Je ne connais pas le nom commercial	Do not know the commercial name
Autre	Other
16. Selon vous, quels sont les inconvénients de la prothèse adjointe flexible ?	16. According to your experience, what are the disadvantages of flexible removable dentures?
Plusieurs réponses possibles	Multiple choices
Déformation pendant la mastication	Deformation during mastication
Surface rugueuse	Rough surface
Détérioration de la couleur	Color deterioration
Difficulté de polissage	Difficulty in polishing
Coût relativement élevé	Relatively high cost
Difficulté de réparation ou de rebasage	Difficulty of repair or relining
Autre	Other
17. D’après votre expérience d’utilisation de la prothèse adjointe flexible, comment jugez-vous les doléances ?	17. According to your experience, how would you rate patient complaints related to flexible removable dentures?
Une seule réponse possible	Single choice
Faibles	Mild
Moyennes	Moderate
Importantes	Severe
18. Vous préférez utiliser la prothèse flexible en cas d’édentements :	18. In which arch do you prefer to use flexible dentures?
Plusieurs réponses possibles	Multiple choices
Maxillaire	Maxillary
Mandibulaire	Mandibular
Quelle que soit l’arcade concernée	Both arches
19. En cas d’édentement partiel de grande étendue, quelle réhabilitation prothétique préférez-vous :	19. In cases of extensive partial edentulism, which prosthetic rehabilitation do you prefer?
Une seule réponse possible	Single choice
La prothèse adjointe flexible	Flexible removable partial denture
La prothèse adjointe en résine conventionnelle	Conventional acrylic removable partial denture
La prothèse adjointe à base métallique (stélitte)+ résine acrylique	Metal-based removable partial denture with acrylic resin
La combinaison châssis+ résine flexible	A combination of metal framework and flexible resin
20. Dans quel cas préférez-vous utiliser la prothèse adjointe flexible ?	20. In which clinical situations do you prefer to use flexible removable dentures?
Plusieurs réponses possibles	Multiple choices
Édentement partiel de grande étendue	Extensive partial edentulism
Édentement partiel encastré	Bounded partial edentulism
Édentement partiel terminal	Distal extension partial edentulism
Risque de visibilité de crochet métallique au niveau antérieur	Risk of visible metal clasps in anterior regions
Édentement complet	Complete edentulism
Autre	Other
21. Comment jugez-vous votre expérience d’utilisation de la prothèse adjointe flexible dans votre cabinet ?	21. How would you evaluate your experience using flexible removable dentures in your practice?
Une seule réponse possible	Single choice
Favorable	Favorable
Défavorable	Unfavorable
22. Chez le patient bruxomane, vous optez pour :	22. In patients with bruxism, which material do you prefer?
Une seule réponse possible	Single choice
La résine acrylique	Acrylic resin
La résine flexible	Flexible resin
23. En présence de contre-dépouilles bilatérales :	23. In the presence of bilateral undercuts:
Une seule réponse possible	Single choice
La résine flexible est préférable par rapport à la résine acrylique	Flexible resin is preferable to acrylic resin
La chirurgie pré-prothétique est indispensable, indépendamment de la résine utilisée	Pre-prosthetic surgery is essential regardless of the type of resin used
